# Value of peak strain dispersion in discovering left ventricular dysfunction in diabetes mellitus

**DOI:** 10.1038/s41598-020-78621-7

**Published:** 2020-12-08

**Authors:** Chunmei Li, Miao Yuan, Kun Li, Wenjuan Bai, Li Rao

**Affiliations:** 1 Department of Cardiology, West China Hospital of Sichuan University, 37 Guo Xue Xiang, Chengdu, 610041 China; 2 Department of Pediatric Surgery, West China Hospital of Sichuan University, Chengdu, 610041 China; 3 Department of Anesthesiology, West China Hospital of Sichuan University, Chengdu, 610041 China

**Keywords:** Type 2 diabetes, Cardiomyopathies

## Abstract

Cardiovascular disease is one of the main causes of death in diabetes mellitus (DM) patients. The aim of the current study was to explore the value of peak strain dispersion (PSD) for discovering early-stage left ventricular (LV) dysfunction in type 2 diabetes mellitus (T2DM) patients. One hundred and one T2DM patients and sixty healthy subjects were selected for this study. T2DM patients were further divided into controlled blood glucose (HbA1c < 7%, n = 46) and uncontrolled blood glucose (HbA1c ≥ 7%, n = 55) subgroups. All participants underwent conventional echocardiography and two-dimensional speckle-tracking echocardiography. Our results showed that an obvious difference was not observed in global longitudinal strain (GLS) between the controlled blood glucose group and the control group (− 20.34% vs − 21.22%, *P* = 0.068). Compared with the healthy controls, the uncontrolled blood glucose group showed an impaired GLS (− 18.62% vs − 21.22%, *P* < 0.001). Nevertheless, PSD was appreciably increased in the controlled blood glucose group (36.02 ms vs 32.48 ms, *P* = 0.01) and uncontrolled blood glucose group (57.51 ms vs 32.48 ms, *P* < 0.001). Multivariate linear regression analysis showed that HbA1c was closely related to PSD lesion in the LV in the T2DM group (β = 0.520, *P* < 0.001). PSD plays an important role in evaluating the coordination and synchronization of myocardial movement and provides a more accurate and sensitive index assessment of early LV systolic function in T2DM patients. In addition, HbA1c levels were related to LV dysfunction.

## Introduction

In recent years, the incidence of type 2 diabetes mellitus (T2DM) has gradually increased. Epidemiological studies show that approximately 70–80% of diabetic patients die of cardiovascular disease, making cardiovascular complications the main cause of death of diabetic patients^1^. In addition, large-sample and multicentre studies have confirmed that compared with nondiabetic patients, diabetic patients have a two- to threefold increase in the risk of congestive heart failure and cardiovascular disease mortality^2^. Furthermore, there are many mechanisms that may lead to myocardial lesion in diabetes, such as myocardial cell metabolism disorder, myocardial fibrosis, myocardial cell apoptosis, microvascular disease, oxidative stress, and inflammatory response^2–4^. Patients with T2DM may have heart function damage to different degrees. Therefore, early detection of cardiac dysfunction and treatment intervention are necessary to reduce the risk and mortality of cardiovascular disease. Left ventricular ejection fraction (LVEF) is the most commonly used index to evaluate left ventricular (LV) systolic function, but a decrease in LVEF often appears in advanced periods of diabetes.

With the rapid development of echocardiography in recent years, the technology can also be used to evaluate early LV systolic dysfunction with myocardial strain index, such as global longitudinal strain(GLS), and global circumferential strain(GCS), obtained by two-dimensional speckle-tracking echocardiography (2DSTE)^5,6^. Accumulating evidence has shown that GLS is the most commonly used strain and can be used to detect early subclinical LV dysfunction in patients with normal LVEF. GLS is significantly reduced in some diseases, such as hypertension^7^ and valvular disease^8^. However, GLS has an inherent shortcoming, ignoring the change in myocardial movement sequence or peak time parameters^9,10^.

The emerging study of peak strain dispersion (PSD) identifies whether the peak time of long axis strain of LV myocardium is consistent, which can directly reflect the effective work of the heart and make up for the deficiency of GLS in evaluating LV systolic function^10–12^. PSD is used to evaluate the early systolic dysfunction of LV by combining the coordination and synchronization of cardiac mechanical movement, and this index has been applied to assess early LV systolic dysfunction in some diseases, such as mitral valve prolapse^13^, aortic stenosis^14^ and hypertrophic cardiomyopathy^15^. In the current study, we aimed to explore the value of PSD for discovering early-stage LV dysfunction in T2DM patients.

## Methods

This study included one hundred and eighteen T2DM patients with New York Heart Association functional classifications of class I or II. All T2DM patients met the diagnostic criteria of diabetes mellitus (DM) according to the 2010 guidelines of the American Diabetes Association^16^_._ Exclusion criteria included coexisting poorly controlled high blood pressure (BP), coronary artery disease, hyperlipidaemia, pericardial disease, cardiac arrhythmia (including atrial fibrillation, branch blocks, etc.), damage to valves (valve (including mitral valve, tricuspid valve, aortic valve and pulmonary valve) regurgitation (mild and above) and any degree of valve stenosis), structural heart diseases or poor acoustic windows. Finally, one hundred and one patients were selected for the case group. The hemoglobin A1c (HbA1c) level is regarded as the most valuable index to judge the state of blood glucose control in patients with T2DM^17^. To evaluate whether controlled blood glucose levels and uncontrolled blood glucose levels may lead to different myocardial lesions in patients with T2DM, the case group was further divided into a controlled blood glucose (HbA1c < 7%, n = 46) group (including 13 cases with hypertension (controlled BP values), 6 cases with obesity, and 6 cases with hypertension (controlled BP values) and obesity) and an uncontrolled blood glucose (HbA1c ≥ 7%, n = 55) group (including 17 cases with hypertension (controlled BP values), 5 cases with obesity, and 8 cases with hypertension (controlled BP values) and obesity ) on the basis of HbA1c levels tested in the past two weeks. Sixty subjects with good ultrasound image quality were also included in the control group in this study. Twenty-eight healthy subjects, 19 controls with hypertension (controlled BP values), 9 controls with obesity and 4 controls with hypertension (controlled BP values) and obesity were included in the control group. All of the subjects of the study provided written informed consent.

### Access to basic clinical data

Age, body surface area (BSA), body mass index (BMI), sex, heart rate (HR), systolic blood pressure (SBP) and diastolic blood pressure (DBP) were recorded for all subjects. Blood samples were obtained from all participants, and the following laboratory tests were performed: high-density lipoprotein (HDL) cholesterol, low-density lipoprotein (LDL) cholesterol, total cholesterol, and triglycerides. HbA1c was also tested, and the duration of T2DM was recorded in patients with T2DM.

### Routine echocardiography

All subjects received routine echocardiography examination. All subjects were in sinus rhythm at the time of echocardiography. Routine echocardiography image acquisition and data measurement were performed using the GE VividE9 ultrasound diagnostic system and M5Sc probe, whose frame rates were adjusted to the range of 1.5–4.6 MHz. LV end diastolic diameter (LVEDD), LV end diastolic volume (LVEDV), peak E, peak A, isovolumetric relaxation time (IVRT), E_m_, and A_m_ were measured in all subjects. LV mass index (LVMI) was assessed by LV mass, which was calculated using the Devereux formula divided by BSA. LVEF was calculated by the biplane Simpson’s method after images were obtained from the 4-chamber and 2-chamber sections of apical views. The E/A ratio, E_m_/A_m_ ratio and E/E_m_ ratio were calculated.

### 2DSTE analyses

**Figure 1:**
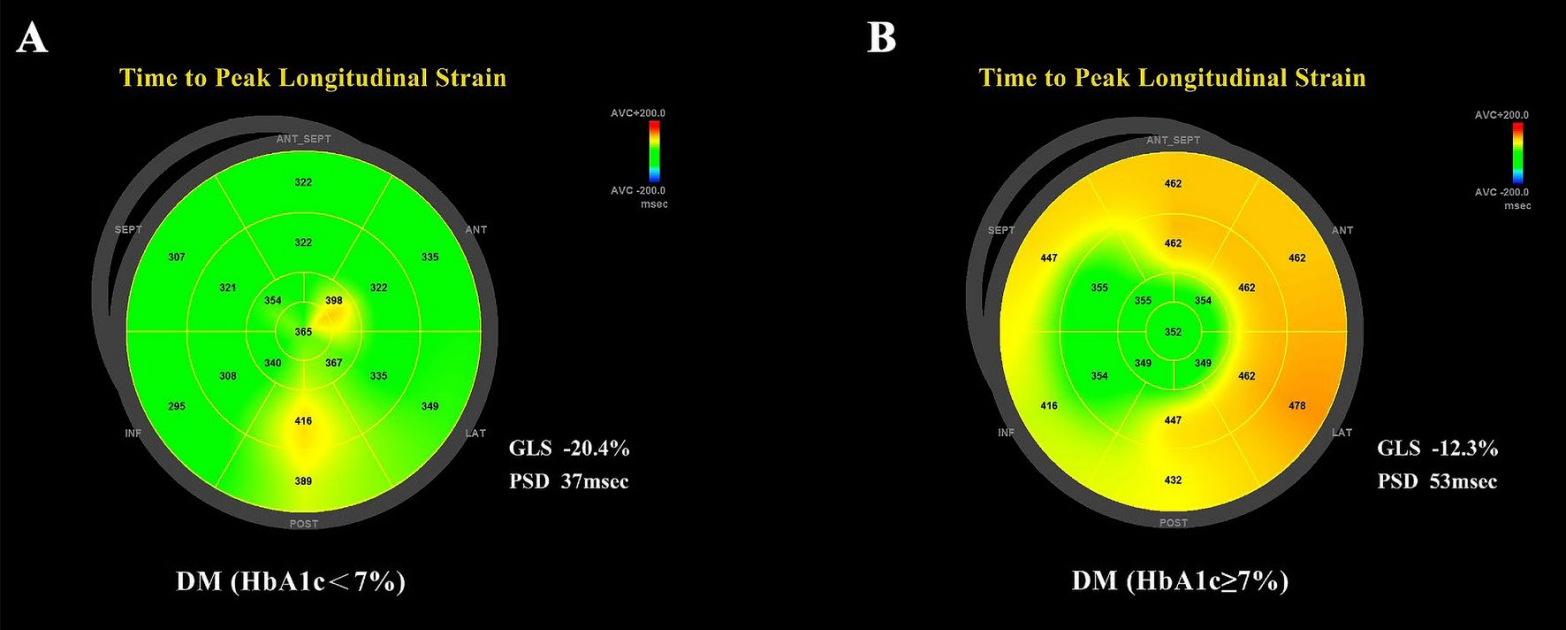
Bullseye plots of time to peak longitudinal strain are shown in two T2DM patients using EchoPAC workstation 2DSTE analysis. The endocardium of the LV was tracked point by point in the apical four-chamber, apical three-chamber and apical two-chamber views. After 17 segments of the LV were successfully tracked, GLS and LV PSD were automatically abtained. A patient with controlled blood glucose (HbA1c < 7%) displayed an LV GLS of 20.4% (absolute value) and LV PSD of 37 ms (**A**). More pronounced LV GLS of 12.3% (absolute value) and LV PSD of 72 ms were observed in a DM patient with uncontrolled blood glucose (HbA1c ≥ 7%) (**B**). *AVC* aortic valve closure, *ANT* anterior, *SEPT* septal, *LAT* lateral, *POST* posterior, *INF* inferior.

Images from 2DSTE were obtained using an M5Sc transducer (Vivid E9) in the four-chamber, three-chamber and two-chamber of apical views for all subjects. All participants were told to hold their breath while images were obtained so that high-quality images could be acquired. When we take images, each patient is connected to an electrocardiograph. All images were collected in 3–5 complete cardiac cycles. The frame rate of the image was fixed in the range of 40–80 frames/s. An advanced quantitative analysis EchoPAC workstation (version 201) was used for 2DSTE analyses. The endocardium of the LV was tracked point by point in the apical four-chamber, apical three-chamber and apical two-chamber views. After 17 segments of the LV were successfully tracked, GLS and LV PSD were automatically abtained. The analysis procedure of GLS and PSD is exhibited in Fig. 1.

### Statistical analysis

All statistical analyses were conducted using SPSS version 23.0 (SPSS, Inc., Chicago, IL). Independent-sample T tests or Mann–Whitney nonparametric tests were applied to compare the clinical and echocardiographic parameters between the case group and the control group. One-way analysis of variance or the Kruskal–Wallis and Mann–Whitney nonparametric tests were employed for comparisons among groups. The normality of continuous data was tested by the Kolmogorov–Smirnov test. Data conforming to a normal distribution are shown as the mean ± SD or as the median (25th and 75th percentiles). The chi-square test was applied to compare categorical data.

To evaluate whether LV dysfunction is affected by multiple factors (including DM duration, HbA1c, age, SBP, LDL, LVMI and E/E_m_ ratio), multivariate linear regression analysis was used to test the risk of the abovementioned factors on GLS and PSD in LV for the case group. *P* values < 0.05 were considered statistically significant for all analyses.

To estimate differences within intra-observer and inter-observer measurements for the GLS and PSD parameters, Bland–Altman analyses were applied to test agreement between the repeated measurements, in which the image acquisition and analysis for all participants were repeated by the same sonographer at distinct times and by a second sonographer on the identical day, respectively. The repeated acquisition and analysis of images was blinded to the previous measurements.

### Ethical approval and study participants

The study protocol was approved by the Ethics Committee of Sichuan University (Sichuan, China). Written informed consent was obtained from all participants. All methods were performed in accordance with relevant guidelines and regulations.

### Consent for publication

All authors give their consent for publication.

## Results

### Basic clinical data for all participants

Table 1 shows the basic clinical data for all subjects. The controlled blood glucose group was similar to the uncontrolled blood glucose group regarding T2DM duration. Compared with the controlled blood glucose group, the uncontrolled blood glucose group had an increased HbA_1_c (9.08% vs 5.74%, *P* < 0.001). There were no considerable differences for age, BSA, BMI, sex, HR, SBP, DBP, HDL, LDL cholesterol, total cholesterol and triglycerides among the groups (*P* > 0.05). Arterial hypertension (controlled BP values) and obesity were equally distributed in the control group and T2DM subgroups.

**Table 1:** Basic clinical data for all participants.

Parameter	Control (n = 60)	T2DM (n = 101)	T2DM_HbA1c<7%_ (n = 46)	T2DM_HbA1c≥7%_ (n = 55)
T2DM duration (years)	NA	7.32 ± 1.85	7.15 ± 1.69	7.45 ± 1.99
HbA_1c_ (%)	NA	7.56 ± 2.20	5.74 ± 0.75	9.08 ± 1.82*
Age (years)	53.83 ± 11.96	52.06 ± 9.04	51.35 ± 7.70	52.65 ± 10.5
BSA (m^2^)	1.63 ± 0.11	1.65 ± 0.12	1.64 ± 0.12	1.66 ± 0.12
BMI (kg/m^2^)	23.03 ± 4.54	24.09 ± 4.10	24.51 ± 3.99	23.75 ± 4.19
Male/female	28/32	47/54	21/25	26/29
HR (beat/min)	75.33 ± 7.55	76.99 ± 8.37	77.35 ± 8.68	76.69 ± 8.17
SBP (mm Hg)	123.22 ± 8.90	123.39 ± 7.35	122.37 ± 8.32	124.24 ± 6.38
DBP (mm Hg)	79.47 ± 5.77	81.13 ± 4.66	80.89 ± 4.35	81.33 ± 4.94
Comorbidities Hypertension n (%)	23 (38.33)	44 (43.56)	19 (41.30)	25 (45.45)
Comorbidities obesity n (%)	13 (21.67)	25 (24.75)	12 (26.09)	13 (23.64)
**Drug therapy n (%)**				
CCB	13 (21.67)	26 (25.74)	15 (32.61)	11 (20.00)
β‐blocker	8 (13.33)	23 (22.77)	12 (26.09)	11 (20.00)
ACEI/ARB	21 (35.00)	37 (36.63)	17 (36.96)	20 (36.36)
Diuretic (including MRA)	0 (0)	0 (0)	0 (0)	0 (0)
**Drug therapy (for DM) n (%)**				
Metformin	NA	53 (52.48)	25 (54.35)	28 (50.91)
Sulfonylurea	NA	19 (18.81)	7 (15.22)	12 (21.82)
Thiazolidines	NA	3 (2.97)	1 (2.17)	2 (3.64)
Nateglinide	NA	21 (20.79)	13 (28.26)	8 (14.55)
α‐GI	NA	16 (15.84)	6 (13.04)	10 (18.12)
DPP-4 inhibitor	NA	43 (42.57)	30 (65.22)	13 (23.64)
GLP‐1RA	NA	0 (0.00)	0 (0.00)	0 (0.00)
SGLT-2 inhibitor	NA	15 (14.85)	10 (21.74)	5 (9.09)
Insulin	NA	19 (18.81)	7 (15.22)	12 (21.82)
HDL (mmol/L)	1.33 ± 0.26	1.27 ± 0.26	1.25 ± 0.25	1.29 ± 0.27
LDL (mmol/L)	2.03 ± 0.53	2.17 ± 0.57	2.12 ± 0.49	2.20 ± 0.63
Total cholesterol (mmol/L)	4.01 ± 0.50	4.17 ± 0.74	4.13 ± 0.72	4.20 ± 0.76
Triglycerides (mmol/L)	1.12 ± 0.34	1.20 ± 0.37	1.19 ± 0.37	1.21 ± 0.37

Data are represented as the mean ± SD or as numbers.

*T2DM* type 2 diabetes mellitus, *HbA*_*1c*_ hemoglobin A_1c_, *NA* not estimated, *BSA* body surface area, *BMI* body mass index, *HR* heart rate, *SBP* systolic blood pressure, *DBP* diastolic blood pressure, *CCB* calcium channel blocker, *ACEI* angiotensin converting enzyme inhibitor, *ARB* angiotensin receptor blocker, *MRA* mineralocorticoid receptor antagonist, *α‐GI* α‐glucosidase inhibitor, *DPP-4* dipeptidyl peptidase 4, *GLP‐1RA* glucagon‐like peptide‐1 receptor agonists, *SGLT-2* sodium-dependent glucose transporter 2, *HDL* high-density lipoprotein, *LDL* low-density lipoprotein.

*p < 0.05 versus control.

^†^*p* < 0.05 versus controlled blood glucose.

### Routine echocardiographic characteristics

Compared with the control group, the T2DM group had a significantly higher LVMI (98.38 g/m^2^ vs 93.55 g/m^2^, *P* = 0.002) and E/E_m_ ratio (9.66 vs 7.66, *P* < 0.001) and an apparently lower E/A ratio (1.38 vs 1.51, *P* = 0.002), E_m_ (9.33 cm/s vs 11.73 cm/s, *P* < 0.001), and E_m_/A_m_ ratio (1.09 vs 1.41, *P* < 0.001). A mild descending peak E (83.05 cm/s vs 87.22 cm/s, *P* = 0.04) and a mild increasing peak A (61.15 cm/s vs 58.28 cm/s, *P* = 0.039) were also observed in the T2DM group matched with the control group. The T2DM and control groups were well matched for IVRT and A_m_. In the comparison among subgroups, we found that, compared with the control group, impaired LVMI and E_m_ were found in the controlled blood glucose group (97.33 g/m^2^ vs 93.55 g/m^2^, *P* = 0.04 for LVMI; 10.86 cm/s vs 11.73 cm/s, *P* = 0.029 for E_m_) and uncontrolled blood glucose group (99.26 g/m^2^ vs 93.55 g/m^2^, *P* = 0.001 for LVMI; 8.05 cm/s vs 11.73 cm/s, *P* < 0.001 for E_m_), but the greatest alteration was found in the uncontrolled blood glucose group. Slightly damaged peak E (81.40 cm/s vs 87.22 cm/s, *P* = 0.012), peak A (61.69 cm/s vs 58.28 cm/s, *P* = 0.033), E/A ratio (1.35 vs 1.51, *P* = 0.001), IVRT (82.02 ms vs 77.97 ms, *P* = 0.019), A_m_ (9.24 cm/s vs 8.58 cm/s, *P* = 0.027), E_m_/A_m_ ratio (0.88 vs 1.41, *P* < 0.001) and E/E_m_ ratio (10.96 vs 7.66, *P* < 0.001) were observed in the uncontrolled blood glucose group. LVEDD, LVEDV and LVEF were comparable among those groups (Table 2).

**Table 2:** Routine echocardiographic characteristics.

Parameter	Controls (n = 60)	T2DM (n = 101)	T2DM_HbA1c<7%_ (n = 46)	T2DM _HbA1c≥7%_ (n = 55)
LVEDD (mm)	45.67 ± 3.06	46.19 ± 3.12	46.37 ± 3.54	46.05 ± 2.75
LVEDV (ml)	99.23 ± 10.83	102.37 ± 11.25	103.13 ± 9.95	101.72 ± 12.29
LVEF (%)	65.02 ± 4.00	63.93 ± 3.97	64.17 ± 4.27	63.73 ± 3.72
LVMI (g/m^2^)	93.55 ± 9.67	98.38 ± 9.09*	97.33 ± 9.44*	99.26 ± 8.78*
Peak E (cm/s)	87.22 ± 12.37	83.05 ± 12.35*	85.03 ± 11.91	81.40 ± 12.57*
Peak A (cm/s)	58.28 ± 7.53	61.15 ± 8.96*	60.50 ± 8.22	61.69 ± 9.58*
E/A ration	1.51 ± 0.25	1.38 ± 0.26*	1.42 ± 0.23	1.35 ± 0.27*
IVRT (ms)	77.97 ± 9.35	80.87 ± 9.10	79.50 ± 8.87	82.02 ± 9.21*
E_m_ (cm/s)	11.73 ± 1.89	9.33 ± 2.49*	10.86 ± 1.94*	8.05 ± 2.16*^†^
A_m_ (cm/s)	8.58 ± 1.91	8.84 ± 1.43	8.35 ± 1.53	9.24 ± 1.22*^†^
E_m_/A_m_ ration	1.41 ± 0.27	1.09 ± 0.36*	1.34 ± 0.33	0.88 ± 0.24*^†^
E/E_m_ ration	7.66 ± 1.72	9.66 ± 3.43*	8.09 ± 1.96	10.96 ± 3.85*^†^

Data are represented as the mean ± SD.

*LVEDD* left ventricular end diastolic diameter, *LVEDV* left ventricular end diastolic volume, *LVEF* left ventricular ejection fraction, *LVMI* left ventricular mass index, *IVRT* isovolumetric relaxation time.

*p < 0.05 versus control.

^†^*p* < 0.05 versus controlled blood glucose.

### Impairment of GLS and PSD parameters in the LV in T2DM patients

**Figure 2:**
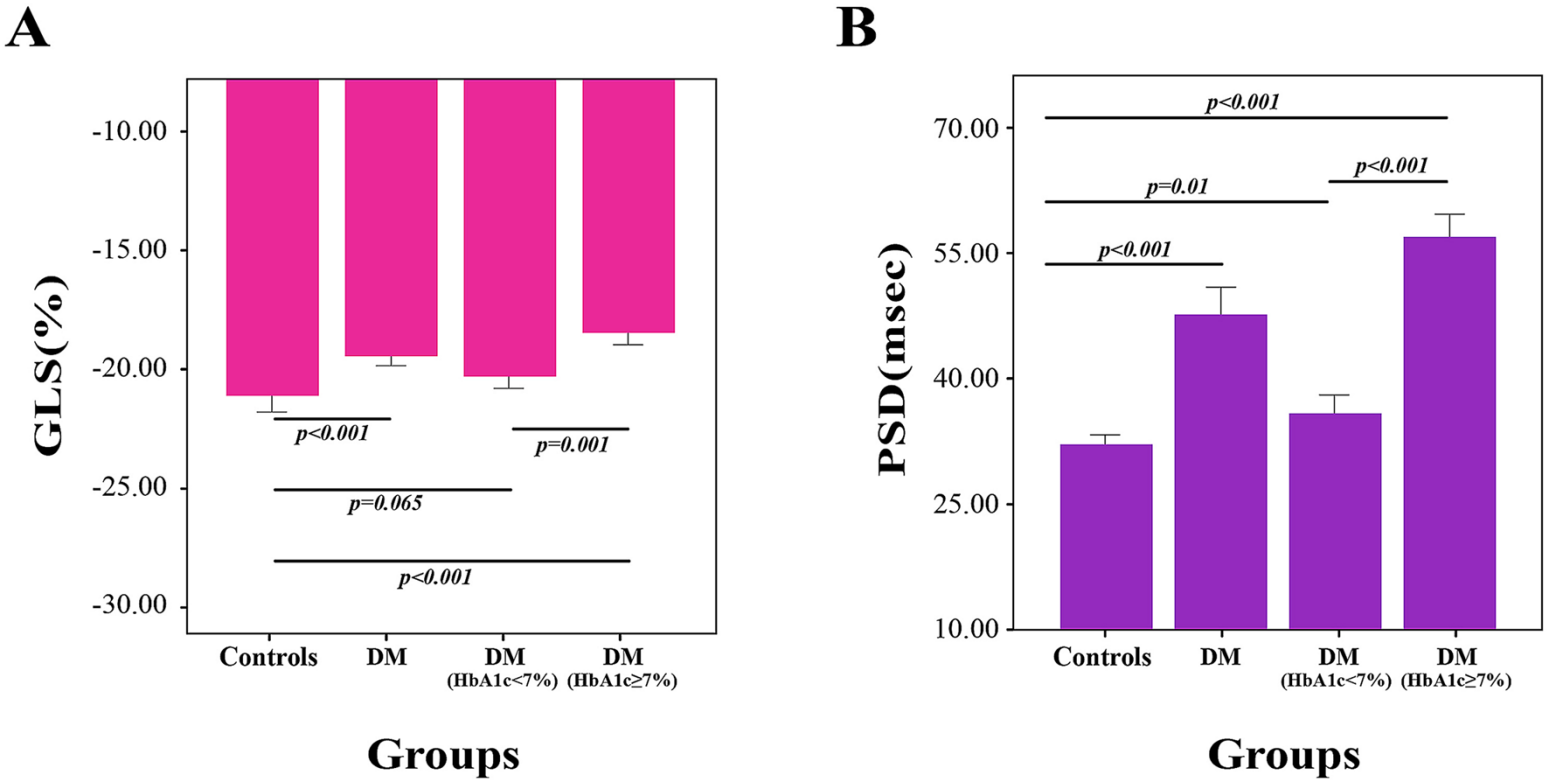
Impairment of GLS (**A**) and PSD (**B**) parameters in the LV in T2DM patients.

GLS (− 19.40% vs − 21.22%, *P* < 0.001), and PSD (47.72 ms vs 32.48 ms, *P* < 0.001) were significantly damaged in the T2DM group. Different changes in GLS and PSD in the controlled blood glucose group and uncontrolled blood glucose group are also displayed in Table 3. Our results showed that an obvious difference was not discovered in GLS between the controlled blood glucose group and the control group (− 20.34% vs − 21.22%, *P* = 0.068). Compared with the healthy controls, the uncontrolled blood glucose group showed an evident impaired GLS (− 18.62% vs − 21.22%, *P* < 0.001). Nevertheless, PSD was appreciably increased in the controlled blood glucose group (36.02 ms vs 32.48 ms, *P* = 0.01) and uncontrolled blood glucose group (57.51 ms vs 32.48 ms, *P* < 0.001). Moreover, a large difference was observed in the uncontrolled blood glucose group (Fig. 2).

**Table 3:** Impairment of GLS and PSD parameters in the LV in T2DM patients.

Parameter	Controls (n = 60)	T2DM (n = 101)	T2DM_HbA1c<7%_ (n = 46)	T2DM _HbA1c≥7%_ (n = 55)
GLS (%)	− 21.22 ± 2.62	− 19.40 ± 2.47*	− 20.34 ± 2.21	− 18.62 ± 2.41*^†^
PSD (ms)	32.48 ± 3.75	47.72 ± 13.56*	36.02 ± 6.24*	57.51 ± 9.69 *^†^

Data are represented as the mean ± SD.

*GLS* global longitudinal strain, *PSD* peak strain dispersion.

*p < 0.05 versus control.

^†^*p* < 0.05 versus controlled blood glucose.

### Risk factors for GLS and PSD values in the LV in T2DM patients

Multivariate linear regression analysis showed that GLS in the LV was affected by HbA1c (β = 0.227, *P* = 0.017), LVMI (β = 0.255, *P* = 0.01) and the E/E_m_ ratio (β = 0.234, *P* = 0.026). HbA1c (β = 0.520, *P* < 0.001), LVMI (β = 0.172, *P* = 0.027) and E/E_m_ ratio (β = 0.289, *P* = 0.001) were correlated with impaired PSD in the LV in the T2DM group. T2DM duration, age, SBP and LDL were not affected by GLS or PSD in the LV. In general, LVMI had an obvious impact on GLS destruction in the LV, and HbA1c was closely related to PSD lesions in the LV in the case group. (Table 4).

**Table 4:** Risk factors for GLS and PSD values in the LV in T2DM patients.

Parameter	β	*P*	GLS 95% CI
T2DM duration (years)	− 0.058	0.510	− 0.307 to 0.153
HbA1c (%)	0.227	**0.017**	0.046 to 0.463
Age(years)	0.068	0.439	− 0.029 to 0.066
SBP(mmHg)	0.073	0.405	− 0.034 to 0.083
LDL cholesterol (mmol/L)	0.124	0.157	− 0.211 to 1.286
LVMI (g/m^2^)	0.255	**0.010**	0.017 to 0.122
E/E_m_ ratio	0.234	**0.026**	0.020 to 0.317

Parameter	β	*P*	PSD 95% CI
T2DM duration (years)	0.036	0.598	− 0.730 to 1.261
HbA_1c_ (%)	0.520	** < 0.001**	2.297 to 4.100
Age (years)	− 0.008	0.907	− 0.219 to 0.194
SBP (mmHg)	0.070	0.314	− 0.124 to 0.382
LDL cholesterol (mmol/L)	− 0.017	0.809	− 3.635 to 2.845
LVMI (g/m^2^)	0.172	**0.027**	0.029 to 0.485
E/E_m_ ratio	0.289	**0.001**	0.500 to 1.784

*Β* standardization coefficient beta, *CI* confidence interval, *T2DM* type 2 diabetes mellitus, *HbA*_*1c*_ hemoglobin A_1c_, *SBP* systolic blood pressure, *LDL* low-density lipoprotein, *LVMI* left ventricular mass index, *GLS* global longitudinal strain, *PSD* peak strain dispersion.

### Reproducibility within intra-observer and inter-observer measurements for the GLS and PSD parameters

**Figure 3:**
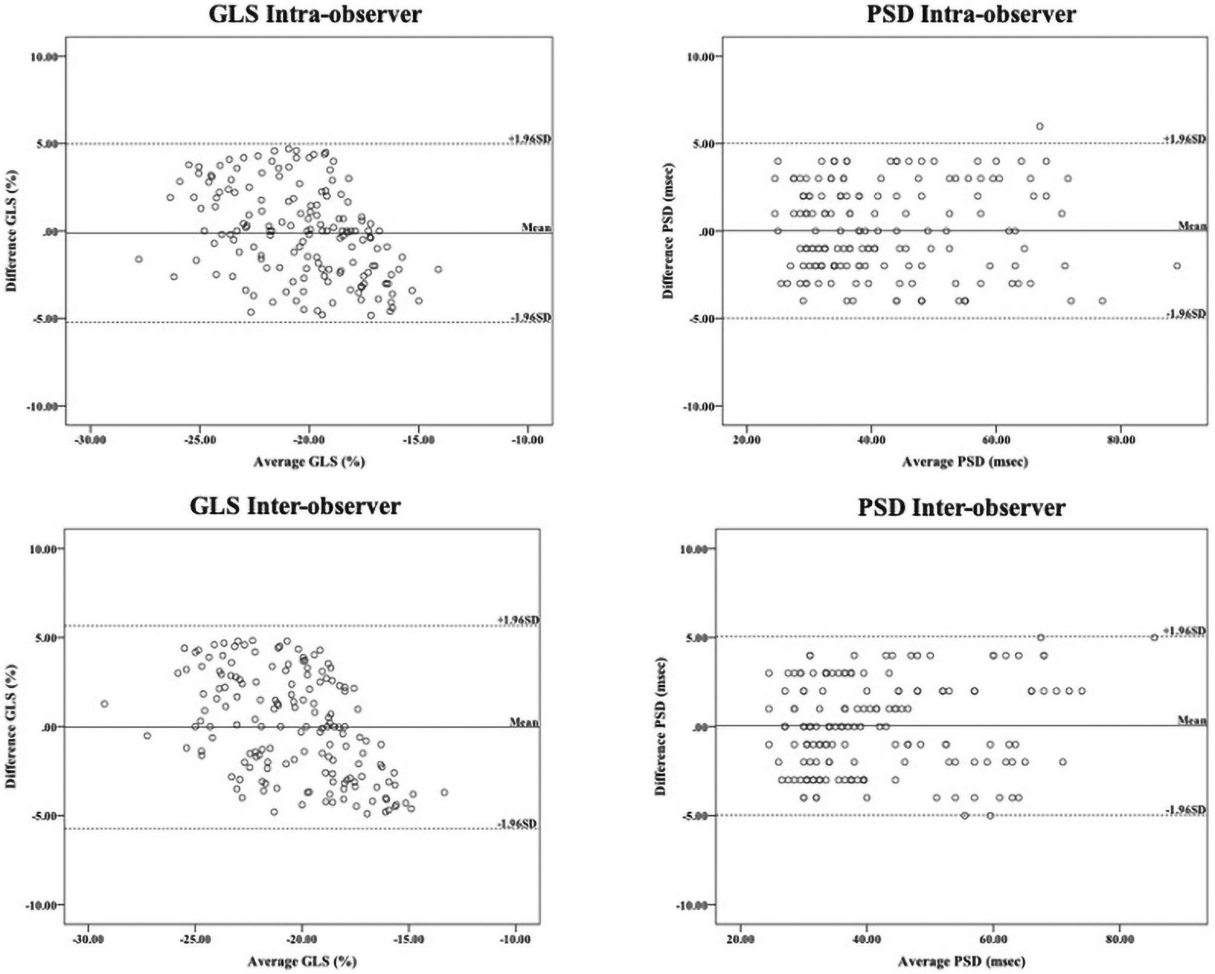
Reproducibility within intra-observer and inter-observer measurements for the GLS and PSD parameters.

Our results showed that the repeatability of intra-observer (GLS for -5.23% to 4.98% and PSD for − 4.99 to 5.01 ms) and inter-observer (GLS for − 5.73 to 5.67% and PSD for − 4.99–5.07 ms) measurements was good (Fig. 3).

## Discussion

Diabetic cardiovascular complications such as myocardial infarction and congestive heart failure, are one of the main causes of death in T2DM patients^1,18^. Therefore, it is important to detect early LV dysfunction and treat it properly in order to reduce the long-term mortality of diabetic patients. Due to its lack of angle dependence, good repeatability and accurate location of myocardial segments, the 2DSTE technique has been confirmed by previous studies: it can be used to identify subclinical LV dysfunction in some diseases with normal LVEF^7,8,19,20^. GLS is the mean value strain that reflects the mechanical motion of 17 segments of the myocardium in the LV. However, effective work of the heart is coupled to the electrical conduction with the mechanical motion of the myocardium. Therefore, accurate assessment of cardiac function should be combined with the coordination and synchronization of myocardium mechanical movement. The newly developed PSD index combines the myocardial deformation and whether the deformation of 17 myocardial segments is uniform in the LV ^10,12^. The above two factors were combined to evaluate LV systolic function. Compared with GLS, PSD is more accurate in evaluating early lesions of LV function. Previous studies also found that PSD was increased in patients with normal GLS and preserved LVEF^13^.

In this study, we found that LVEF was comparable between the control group and the T2DM group. The LVMI and E/E_m_ ratio were obviously increased in the T2DM group compared with the control group. The T2DM group had a significantly lower E/A ratio, E_m_ and E_m_/A_m_ ratio. More importantly, GLS and PSD were evidently destroyed in the T2DM group. Our results demonstrated that (1) GLS and PSD may be used to detect early LV systolic dysfunction in T2DM patients with preserved LVEF, (2) the LV myocardium is damaged, and the coordination and synchronization of myocardial movement are poor in T2DM patients, (3) subclinical LV diastolic and systolic function were impaired in patients with T2DM, and (4) LV remodelling and hypertrophy may occur in patients with T2DM. These phenomena may be explained by the following reasons. Various pathogeneses, such as metabolic disorders, cardiomyocyte apoptosis, microvascular disease, oxidative stress, and mitochondrial structural disorders, may be involved in myocardial hypertrophy and compliance reduction, LV remodelling and ventricular wall rigidity^21,22^, which may lead to damage to LV systolic and/or diastolic function. Therefore, PSD by 2DSTE may be used to estimate subclinical LV dysfunction, which is consistent with previous studies^13–15,23^.

HbA1c is the most valuable index to judge the state of blood glucose control. The index is stable and can be used to reflect the average blood glucose level in the two months before blood sampling^17^. Therefore, to explore the relationship between blood glucose level and LV dysfunction, we divided T2DM patients into two subgroups (including the controlled blood glucose group and uncontrolled blood glucose group) based on HbA1c level. The outcomes of the present study showed that there was no difference in LVEF among those groups. Damaged LVMI and E_m_ were observed in the two subgroups compared with the control group, but the most obvious change was seen in the uncontrolled blood glucose group. Impaired peak E, peak A, E/A ratio, IVRT, A_m_, E_m_/A_m_ ratio and E/E_m_ ratio were rarely observed in the uncontrolled blood glucose group. In addition, we also observed that GLS was not different between the controlled blood glucose group and the control group. The uncontrolled blood glucose group matched with healthy controls had a significantly impaired GLS. However, PSD was evident in the two subgroups. A noticeable difference was found in the uncontrolled blood glucose group. Our results showed that (1) PSD is a more sensitive and accurate indicator than GLS in the detection of early LV systolic dysfunction. PSD estimates whether the peak time of long axis strain of the LV myocardium is consistent ^10–12^. Recent paper has also shown that PSD is associated with an elevated risk of fatal arrhythmias and sudden cardiac death in patients with coronary artery disease and hypertension^23^. Thus, compared with GLS, PSD may be used to evaluate early LV dysfunction more comprehensively by combining the coordination and synchronization of cardiac mechanical movement. (2) There are still inconsistencies in the synchrony and coordination of myocardial movement in patients with T2DM with well-controlled blood glucose. (3) Cardiac dysfunction was more obvious in the poor blood glucose control group with T2DM.

In addition, the results of this study also showed that HbA1c, LVMI and the E/E_m_ ratio were related to GLS lesions and PSD in the LV in the case group. The LVMI has an evident influence on impaired GLS in the LV, and HbA1c is closely correlated with destroyed PSD in the LV. These results indicated that the more hypertrophic the LV and the higher the level of HbA1c, the more serious the damage to LV function, which corresponds to previous reports. The reasons may be as follows: (1) Hyperglycaemia may generate excessive reactive oxygen species through the electron transport chain, which may result in myocyte apoptosis^24^. (2) Hyperglycaemia may induce other biochemical cascade reactions of myocardial injury, stimulate the expression and accumulation of collagen and promote the cross connection of collagen^25^, which may lead to increased myocardial fibrosis and decreased myocardial compliance. (3) Increased glucose uptake of myocardial cells may affect their energy metabolism, which may lead to the occurrence of diabetic cardiomyopathy and obvious damage to myocardial function^26^. (4) Myocardial compensatory hypertrophy may occur after myocardial damage, which may result in limitations of myocardial movement and damage to myocardial deformation^4^.

Overall, PSD plays an important role in evaluating the coordination and synchronization of myocardial movement and provides a more accurate and sensitive index assessment of early LV systolic function in T2DM patients. Lesions of GLS appear more frequently in T2DM patients with poor blood glucose control. PSD is more susceptible to early estimation of LV systolic dysfunction in T2DM patients with well-controlled blood glucose. In addition, HbA1c level, LVMI and E/E_m_ were all related to damaged LV function. The higher the level of blood sugar is, the greater the damage to heart function. Therefore, early monitoring of cardiac function and active control of blood glucose are of great clinical significance for improving prognosis and reducing mortality for T2DM patients.

## Research limitations

The limitations of this study include the following. First, the study was a cross-sectional study, lacked follow-up data, and failed to evaluate the prognostic significance of damaged GLS and PSD in T2DM patients; Second, the study was a single-centre study with a small sample size. Third, the study evaluated GLS in the LV, and layer-specific analysis should be considered in future studies. Finally, 2DSTE requires high image quality, and some patients were not included because of poor image quality.
